# Codon bias and the folding dynamics of the cystic fibrosis transmembrane conductance regulator

**DOI:** 10.1186/s11658-016-0025-x

**Published:** 2016-10-19

**Authors:** Rafal Bartoszewski, Jaroslaw Króliczewski, Arkadiusz Piotrowski, Anna Janaszak Jasiecka, Sylwia Bartoszewska, Briana Vecchio-Pagan, Lianwu Fu, Aleksandra Sobolewska, Sadis Matalon, Garry R. Cutting, Steven M. Rowe, James F. Collawn

**Affiliations:** 1 Department of Biology and Pharmaceutical Botany, Medical University of Gdansk, Hallera 107, 80-416 Gdansk, Poland; 2 Laboratory of Chemical Biology, Faculty of Biotechnology, University of Wroclaw, Wroclaw, Poland; 3 Department of Inorganic Chemistry, Medical University of Gdansk, Gdansk, Poland; 4 Institute of Genetic Medicine, Johns Hopkins University School of Medicine, Baltimore, USA; 5 Department of Anesthesiology and Perioperative Medicine, University of Alabama at Birmingham, Birmingham, USA; 6 Department of Cell, Developmental, and Integrative Biology, University of Alabama at Birmingham, Birmingham, USA; 7 Departments of Medicine and Pediatrics, University of Alabama at Birmingham, Birmingham, USA; 8 Gregory Fleming James Cystic Fibrosis Center, University of Alabama at Birmingham, Birmingham, USA

**Keywords:** Synonymous mutations, Single nucleotide polymorphism (SNP), Codon usage, mRNA folding, Translation rate, *in silico* predictions, CFTR

## Abstract

Synonymous or silent mutations are often overlooked in genetic analyses for disease-causing mutations unless they are directly associated with potential splicing defects. More recent studies, however, indicate that some synonymous single polynucleotide polymorphisms (sSNPs) are associated with changes in protein expression, and in some cases, protein folding and function. The impact of codon usage and mRNA structural changes on protein translation rates and how they can affect protein structure and function is just beginning to be appreciated. Examples are given here that demonstrate how synonymous mutations alter the translational kinetics and protein folding and/or function. The mechanism for how this occurs is based on a model in which codon usage modulates the translational rate by introducing pauses caused by nonoptimal or rare codons or by introducing changes in the mRNA structure, and this in turn influences co-translational folding. Two examples of this include the multidrug resistance protein (p-glycoprotein) and the cystic fibrosis transmembrane conductance regulator gene (CFTR). CFTR is also used here as a model to illustrate how synonymous mutations can be examined using *in silico* predictive methods to identify which sSNPs have the potential to change protein structure. The methodology described here can be used to help identify “non-silent” synonymous mutations in other genes.

**Electronic supplementary material:**

The online version of this article (doi:10.1186/s11658-016-0025-x) contains supplementary material, which is available to authorized users.

## Background

### How silent polymorphisms can alter protein function

Synonymous single nucleotide polymorphisms (sSNPs) are a common feature of the human genome, yet they are often overlooked in genetic analyses unless they are near mRNA splice junctions. Nowadays, however, it has become increasingly clear that synonymous mutations affect a number of other important cellular processes including mRNA stability, and protein expression, folding and function [1–5]. While the role of synonymous mutations in splicing defects is discussed elsewhere [4], here we focus on how they influence these other processes. Our approach is to cite a number of examples that illustrate how synonymous mutations affect protein expression and function, and then discuss how *in silico* methods can be used to help identify these types of mutations.

Synonymous mutations have been shown to have effects in a number of diseases and disorders including amyotrophic lateral sclerosis [6], increased pain sensitivity [7], melanoma [8], schizophrenia [9], congenital heart disease [10], drug resistance [1], and cystic fibrosis [11–13]. The details describing how these synonymous mutations have their effects are described below.

Amyotrophic lateral sclerosis (ALS) or Lou Gehrig’s disease is a rapidly progressive fatal neurological disease with unclear etiology, although mutations in copper/zinc superoxide dismutase (SOD1) are often associated with this disease. Michael Strong and colleagues demonstrated that wild type *SOD1* mRNA forms ribonucleoprotein complexes with protein homogenates of neuronal tissues that stabilize the *SOD1* mRNA, whereas mRNAs containing ALS missense mutations fail to form these complexes and subsequently have less stable mRNA [6]. More interestingly, 4 silent mutations that have been identified in ALS, Gly11 (C/T) [14], Ser60 (T/C; rs373888553) [15], Thr117 (A/G) [16], and Ala141 (T/A; rs143100660) [15], and all of these fail to form the ribonucleoprotein complexes in a manner similar to that seen for the missense mutations [6]. The results from these studies indicate that loss of ribonucleoprotein binding results in a loss of mRNA stability. This illustrates an interesting and unexpected mechanism for how a synonymous mutation can affect protein expression levels.

Another example is found in the catechol-*O*-methyltransferase (*COMT*) gene. COMT regulates pain perception and there are three haplotypes of the *COMT* gene that are associated with pain perception and developing temporomandibular joint disorder [17]. The three haplotypes are made up of four SNPs, one located in the promoter region, and the other 3 in coding regions. Two are synonymous mutations, one at His62 (C/T; rs4633) and another at Leu136 (C/G; rs4818), and the third is a missense mutation, Val158Met (A/G; rs4680) [7]. Interestingly, the synonymous changes account for the largest change in enzyme activity [7]. Diatchenko and colleagues focused on understanding how these synonymous mutations caused this loss of protein expression and suggested that the differences were associated with mRNA secondary structural differences. To test this idea, they analyzed the mRNA secondary structures with the predictive algorithms Mfold [18] and Afold [19]. This study also illustrated that the *in silico* predictive methods for mRNA folding were confirmed by using site-directed mutagenesis to destabilize the predicted stem-loop structures. This manipulation destabilized the predicted mRNA structures and resulted in an increase in protein and enzyme activities, establishing that very stable mRNA secondary structures or those less likely to unfold easily during translation are associated with less translated protein [19].

Even in the cancer genomics field, the role of synonymous mutations is now beginning to be appreciated. Yardena Samuels and colleagues identified somatic mutations in 29 melanoma samples and found an interesting synonymous mutation in the Bcl-2-like protein 12 (*BCL2L12*) gene that is as an anti-apoptotic factor (a C to T change at position 51 (F17F)) [8]. This mutation leads to increased *BCL2L12* mRNA and protein levels. In characterizing this silent mutation, they found that this mutation occurred in 10 of 256 melanomas and that the elevations in mRNA and protein were not due to splicing or translation changes or to changes in protein stability [8]. They found that the mutation causes an accumulation in mRNA and protein and this promoted antiapoptotic signaling in the melanoma cells [8]. Analysis of the mechanism involved revealed the surprising finding that this synonymous mutation elevated mRNA levels because of the differential targeting of wild type and mutant BCL2L12 by hsa-miR-671-5p [8]. Interestingly, this type of effect was seen previously between synonymous mutations and altered miRNA binding in the immunity-related GTPase family M (*IRGM*) gene in Crohn’s disease [20].

An extremely thorough and interesting examination of the multidrug resistance 1 (*MDR1*) gene indicated that a synonymous SNP in this gene altered the drug and inhibitor interactions in the gene product, P-glycoprotein [1]. P-glycoprotein is an ATP-driven efflux pump that contributes to the multidrug resistance of cancer cells. One particular synonymous SNP, C3435T, that was a part of a common haplotype was associated with altered P-glycoprotein activity, and Chava Kimchi-Sarfaty analyzed this mutation in detail in a broad range of cell lines and found no change in the level of mRNA or protein in cells expressing this sSNP [1]. Their results, however, indicated that this sSNP introduced a rare codon that altered the protein structure and function, suggesting that translation was altered in the presence of the rare codon [1].

Perhaps the biggest reason that synonymous mutations are often overlooked is that the vast majority of them, at least in most cases, are functionally neutral. In a study on the human dopamine receptor D2 gene (*DRD2*), however, Gejman and colleagues examined the functional properties of six known naturally occurring synonymous mutations and surprisingly found that two had functional effects [9]. C957T was predicted to alter the mRNA folding and this affected the mRNA stability and translation, and importantly, a weakened response to dopamine-induced up-regulation of *DRD2* [9]. The other synonymous mutation, G1101, did not have an effect on its own, but did block the effects of the C957T mutation in the compound clone C957T/G1101A, demonstrating that compound synonymous mutations can have unexpected consequences [9]. Given that dopamine receptors are drug targets in the therapies of schizophrenia, Parkinson’s and Huntington’s diseases [9], the importance in analyzing synonymous mutations in this gene are obvious.

In human congenital heart disease, there are known mutations in cardiac-specific transcription factor genes that impact protein function and the *NK2 transcription factor related, locus 5* gene (*NKX2-5*) provides a good example [10] of this. In *NKX2-5*, more than 40 mutations have been identified in congestive heart failure patients. In a recent study by Jurgen Borlak and colleagues, they analyzed cardiac biopsies of 28 patients and identified a missense mutation in the *NKX2-5* gene, A119E, along with two synonymous mutations in-cis, c.543G > A (Q181Q) and c.63A > G (E21E) [10]. In vitro functional analyses of the transcriptional activities of *NKX2-5* using reporter plasmids revealed that the A119E mutation resulted in as much as a 40 % reduction in activity, and the addition of one or two synonymous mutations reduced the transcriptional activities even further, suggesting that the synonymous mutations exacerbated the phenotype of the missense A119E mutation [10]. Furthermore, using the Vienna RNA folding algorithm for predicting mRNA structure, the authors found that the mRNA secondary structure A119E mutant differed from wild type mRNA and that the addition of the two synonymous mutations changed the structure even further [10]. These studies suggest that in some cases, synonymous mutations, while perhaps not causal in and of themselves, can exacerbate the effects of a missense mutation [10].

## The cystic fibrosis transmembrane conductance regulator (*CFTR*) gene and the *F508del* mutation

Prior studies by Bebok and colleagues found a similar effect in the *CFTR* gene [12]. We examined a synonymous mutation in the most common mutation in the *CFTR* gene, *F508del*, an out-of-frame deletion of phenylalanine that creates a synonymous mutation for isoleucine at position 507 [12]. The human *CFTR* gene is particularly interesting given that it codes for a protein that is highly sensitive to co-translational folding [1, 21–23]. CFTR is a chloride and bicarbonate channel and key regulator of epithelial functions [24–27]. Mutations in the *CFTR* gene lead to reduced or dysfunctional CFTR protein and cause cystic fibrosis (CF), a generalized exocrinopathy affecting multiple organs, but is most notably associated with lung disease [28].

**Fig. 1:**
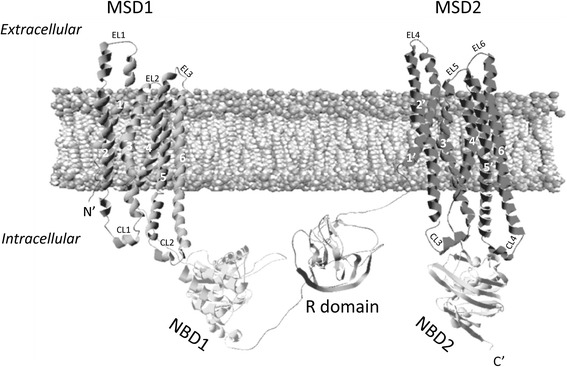
A schematic model of the proposed structure of the cystic fibrosis transmembrane conductance regulator (CFTR) using RasMol 2.7.5.2 (http://www.openrasmol.org) based on the RSCB PDB database coordinates deposited for the human CFTR. The domains model are based on the data published by [57]

The CFTR protein consists of a modular structure composed of two membrane-spanning domains (MSD1 and MSD2, each comprising six transmembrane regions), two nucleotide binding domains (NBD1 and NBD2), and a unique domain among ATP-binding cassette (ABC) transporters called the regulatory domain (R) (Fig. 1) [24]. NBD1 and NBD2 participate in ATP binding and hydrolysis, while phosphorylation of the R domain regulates channel gating [24]. Achieving the proper conformation of the individual domains and interactions between these domains during protein synthesis is critical for proper CFTR assembly [21, 22, 29, 30].

In order to reach its native tertiary structure, CFTR molecules undergo complex hierarchical folding processes and posttranslational modifications [31–33]. The rate of wild type and F508del CFTR translation in transfected human embryonic kidney (HEK 293) cells has been calculated to be 2.7 residues per second based on an average translation rate of 9.2 min [34]. This is slower compared to the average translation rate for other proteins of 4–5 residues per second [35], suggesting that the CFTR translation rate is unusual [21]. CFTR folding begins co-translationally [21], and is completed post-translationally [22, 31, 32]. Du et al. (2009) suggest that the individual domains achieve loosely folded conformations co-translationally, but the final native tertiary structure requires completion of the proper domain interactions [22]. CFTR domain assembly has been estimated to take ~30–120 min [36].

Since CFTR translation and folding occur simultaneously, the choice of codons can affect the translational kinetics during protein synthesis, but how that is linked to protein folding is just beginning to be appreciated [37]. Translational kinetics are believed to be controlled, at least in part, by optimal and non-optimal codons (reviewed in [37]). Optimal codons are postulated to be translated faster, whereas non-optimal are translated slower, with non-optimal codons strategically placed to slow translation and promote co-translational folding. Given the co-translation folding of CFTR and its slow translation rate, we investigated how codon usage is predicted to influence CFTR’s translational rate based on its utilization of optimal and non-optimal codons, and how these changes are predicted to affect the co-translational folding within the individual domains of CFTR. We also analyzed the known CFTR sSNPs that have been identified in order to predict how they might affect the CFTR translational kinetics, mRNA structure, and the co-translational protein folding changes.

## *In silico* predictive methods for identifying synonymous mutations that impact protein function

Highly expressed genes often contain codons that are recognized by the most abundant tRNAs and are considered optimal or fast since they are translated faster [38]. CFTR, on the other hand, is expressed at extremely low amounts, and the translation rate appears to be slower than average [34]. Complex proteins generally utilize rare codons that often localize at strategic domain-domain interfaces [39], and these rare codons (or clusters of rare codons) promote ribosome pauses that may contribute to changes in the folding pathways [39]. Since CFTR is a complex and multi-domain transmembrane protein with distinct transmembrane and cytoplasmic regions, we examined the composition of codons used in human CFTR and determined whether the codons were optimal or rare, and how their placement corresponded to predicted secondary structures and CFTR domain organization.

**Fig. 2:**
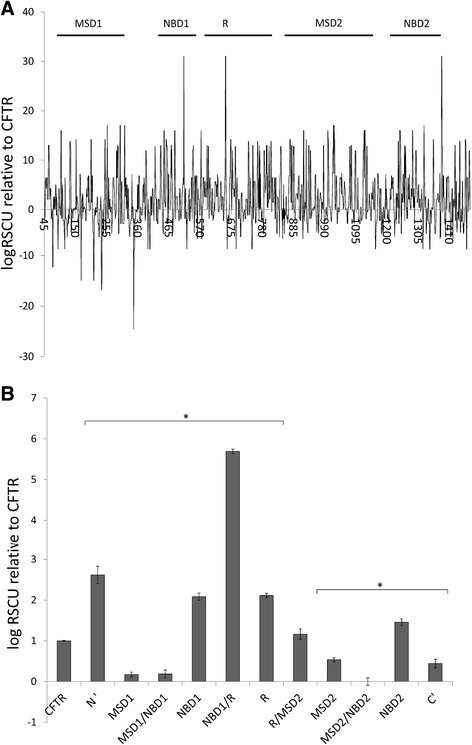
**a** The distribution of optimal and rare codons in CFTR. The logarithm transformed moving median of RSCU values (3-amino acid window) suggests the presence of slow/nonoptimal (negative log RSCU values) and fast/optimal (positive) translated patches within the CFTR primary structure. The amino acid medians were normalized to whole CFTR median RSCU (value 1). The CFTR domain location is marked above the graph. **b** CFTR domains are translated with different rates as shown by their median RSCU values. The domain medians were normalized to whole CFTR median RSCU (value 1). Significantly faster (>1) and slower (<1) translation of the domains are marked with an *, while error bars represent the standard error of the mean (SEM)

In order to identify the predicted fast and slow translating regions in CFTR, we used the relative synonymous codon usage (RSCU) method [40–43] to calculate the potential codon impact on the translation rate (for methods, see Additional file 1). The analysis reveals that CFTR’s codon bias clearly consists of fast and slow translating regions, while the N-terminal transmembrane MSD1 domain shows the highest content of slow translating codons (Fig. 2a, negative log RSCU numbers, that were compared to the entire CFTR molecule that was normalized to 1). This is particularly evident at the end of MSD1, which is critical to endoplasmic reticulum-associated degradation (ERAD) escape and also the location responsible for binding to the CFTR misfolding corrector drug, VX-809 [44]. The log RSCU of the individual CFTR domains is shown in Fig. 2b. All of these regions were compared to the entire CFTR molecule that was normalized to 1. The results shown in Fig. 2b indicate that transmembrane MSD1 and MSD2 are predicted to be the slowest translated regions in CFTR (~5 fold slower than the mean rate of CFTR). Other regions predicted to be translated slowly include the sequences between MSD1 and NBD1 (MSD1/NBD1) and between MSD2 and NBD2 (MSD2/NBD2), and the carboxy-terminal tail region (C’). The RSCU predictions also indicate that the N terminal region (N’), NBD1 and NBD2, the region between NBD1 and the R domain (NBD1/R), and the R domain are translated significantly faster than the CFTR average. The sequence between NBD1 and R domain has the highest log RSCU value in CFTR (Fig. 2b). These data predict that CFTR translation starts relatively fast, slows down while forming the MSD1 domain and then speeds up again during synthesis of the NBD1 and R domains. The MSD2 domain translation is slow, and the slowest predicted translational rate is at the interface between MSD2 and NBD2 (MSD2/NBD2). After this, the translation of NBD2 is predicted to proceed quickly again, before slowing down again at the C-terminus (C’) (Fig. 2b).

**Fig. 3:**
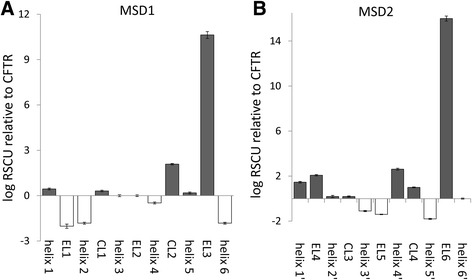
The relative translation rate of the subdomains within MSD1 (**a**) and MSD2 (**b**). The subdomains of MSD1 and MSD2 medians were normalized to whole CFTR median RSCU (value 1)

Both transmembrane domains (MSD1 and MSD2) are composed of membrane spanning alpha helices connected by extracellular and cytosolic loops. Since both of these regions appear to be translated more slowly than average for CFTR, we examined these regions in more detail. As shown in Fig. 3a, almost the entire MSD1 is predicted to be composed of relatively slow translating codons except for cytosolic loop 2 (CL2) and external loop 3 (EL3). The slowest translating regions of MSD1 are helices 2 and 6 and external loop 1 (EL1) (Fig. 3a). The MSD2 codon bias is similar to MSD1 except that the prediction is that helices 1’ and 4’ and external loops 4 and 6 (EL4 and EL6) are translated faster than their counterparts in MSD1 (Fig. 3b). Interestingly in both MSDs, the prediction is that the final external loops are translated very rapidly (EL3 and EL6), while the final helices are translated very slowly (helices 6 and 6’), suggesting that the slowdown in translation is important at the end of both of these transmembrane domains.

## Codon usage effects on the translational kinetics

There are 128 reported sSNPs in the human CFTR mRNA sequence and we analyzed the RSCU value changes introduced by these sSNPs to see how these might affect the translational kinetics (Additional file 2: Table S1). The median ΔRSCU value for the SNPs was 0.075, and almost one-third of those analyzed (42 sSNPs) introduced either significantly higher or lower RSCU values (highlighted in light grey in Additional file 2: Table S1). To determine if these sSNPs were predicted to alter translational rate, we analyzed each sSNP in 3–, 5-, and 10-amino acid RSCU windows as described in the Additional file 1. As shown in Table 1, only 5 of the selected sSNPs (shown in bold in Table 1) were predicted to alter the translational rate (one standard derivation above or below the median for all 3 RSCU window analyses: c.1098A > G (G366 at the MSD1/NBD1 interface); c1641A > T (*T547* in NBD1); c.3472C > A (R1158 at the MSD2/NBD2 interface); c.3772 T > C (L1258 in NBD2); and c.3789 T > C (T1263 in NBD2) (Table 1). The other sSNPs listed in Table 1 showed effects in at least 2 of the 3 RSCU windows, and depending on their location, could potentially affect the CFTR translational kinetics.

**Table 1:** sSNPs selected significant change in local codon bias motifs analyzed for 3-, 5- and 10-aa clusters

sSNP	CFTR domain	Impact on CFTR translation	Significant RSCU change when analyzed in		
			3 aa	5 aa	10 aa
c.333G > A (P111)	MSD1 (EL1)	↑	−	+	+
c.612 T > G (A204)	MSD1 (helix 3)	↓	+	−	+
c.888 T > C (T296)	MSD1 (CL2)	↓	+	+	−
c.981A > G (L327)	MSD1 (helix 5)	↑	+	−	+
c.1074A > G (V358)	MSD1/NBD1	↑	+	+	−
c.1098A > G (G366)	MSD1/NBD1	↓	+	+	+
c.1164G > T (T388)	MSD1/NBD1	↑	+	+	−
c.1641A > T (T547)	NBD1	↓	+	+	+
c.1734A > G (L578)	NBD1	↑	+	−	+
c.2241G > A (A747)	R	↑	−	+	+
c.2373A > G (T791)	R	↓	+	−	+
c.2805A > G (L935)	MSD2 (CL3)	↑	+	+	−
c.2907A > G (A969)	MSD2 (CL3)	↓	+	+	−
c.3472C > A (R1158)	MSD2/NBD2	↑	+	+	+
c.3772 T > C (L1258)	NBD2	↑	+	+	+
c.3780A > G (L1260)	NBD2	↑	−	+	+
c.3789 T > C (T1263)	NBD2	↓	+	+	+
c.3897A > G (T1299)	NBD2	↓	+	+	−

## Role of sSNPs in mRNA structural changes

The sSNP location within the domain, however, may not be the only decisive factor that affects protein folding. Here we examine how codon usage changes could potentially alter mRNA structure given that mRNA structural changes can affect the translational rate and protein function [12, 13]. Using RNAsnp software [45, 46] to determine if any of the SNPs could potentially influence CFTR mRNA structure (Additional file 3: Table S2), 8 SNPs were identified as potential candidates. All of the identified 8 SNPs introduced changes in the mRNA secondary structure by introducing hairpin turns, or by reorganizing or removing them (shown in Additional file 4: Figure S1).

## Predicted pathogenicity of the sSNPs using CADD

In the third type of analysis, we used a recently described tool for estimating the relative pathogenicity of human genetic variants based on the combined annotation dependent depletion (CADD) method [47]. CADD estimates the relative pathogenicity of variants based on annotations from a variety of sources and combining them into a single measure that is expressed as a C-score [47]. The calculated C-scores resemble results from both conservation-based metrics and subset-relevant functional metrics. Interestingly, the sSNP distribution in *CFTR* is characterized by significantly higher average C-scores than observed in whole genome SNP distribution (Additional file 5: Figure S2) [47]. Examining this for *CFTR*, we considered only C-score values that were significantly higher than whole genome sSNP mean (6.36866 + 3.701498 = 10.07) [47], and this led to the initial selection of 47 SNPs. (Additional file 5: Figure S2; Additional file 6: Table S3). Interestingly, 4 of these sSNPs also showed up in the mRNA structural changes analyses, and one of these showed up in all 3 analyses, c.3472C > A (R1158), that is in the MSD2/NBD2 interface (Table 2).

**Table 2:** sSNPs selected as significant by at least 2 independent analysis types (RSCU, mRNA structure and CADD)

sSNP	CFTR domain	RSCU	mRNA structure	CADD
c.612 T > G (A204)	MSD1 (helix 3)	+	+	−
c.3345C > A (T1115)	MSD2 (helix 5′)	−	+	+
c.3472C > A (R1158)	MSD2/NBD2	+	+	+
c.3504C > T (D1168)	MSD2/NBD2	−	+	+

## Translation rates and protein folding

CFTR fits well into the classic model of protein folding that suggests that the transmembrane regions are translated slower than cytosolic regions. Indeed, if one takes a closer look at the composition of the CFTR transmembrane domains (Fig. 3), it is clear that the helixes are formed very slowly, particularly as the amino acids leave the transmembrane region. MSD2 is formed slightly faster than MSD1 and both of these domains share common features. The last external loops of these transmembrane domains (EL3 and EL6) contain optimal codons whereas the last helices (6 and 6′), as well as the interface between the membrane spanning domains and the NBDs (MSD1/NBD1 and MSD2/NBD2) are composed of rare codons. It is clear that MSD1 formation and cytosolic loop assembly are crucial for both co- and posttranslational CFTR folding. Hence, the changes in codon bias in these regions, especially at helices entering or leaving the membrane introduced by mutations or sSNPs could affect CFTR folding efficiency significantly and thus the levels and/or function of the mature protein.

Translation pauses are a general strategy that is employed in the co-translational folding of individual domains in a multi-domain protein. The time separation provided by the pause allows completion of the processes without interruption, thus helping to avoid problems in protein folding and aggregation [48]. Interfering with this process with changing codons introduced by sSNPs can lead to downstream effects. For example, changing codons has been shown in a number of cases to alter protein structure and function [1, 3, 39, 49, 50]. SNPs have been proposed to lead to alternate folding pathways through ribosome stalling, a lower concentration of cognate tRNAs (codon usage), or through alteration of the RNA structure [12, 13, 39, 51]. Altered RNA secondary structures have been shown to influence the length of the pause cycles and the rate of translation [52]. Thus, mRNA structural-related changes in translational dynamics likely influence membrane integration and co-translational folding of multi-spanning membrane proteins like *CFTR* [21, 53]. Our previous studies demonstrate that mRNA structural changes associated with the I507 SNP introduced by the F508del CFTR mutation results in a decreased translational rate of *F508del CFTR* [12]. Furthermore, a synonymous single nucleotide variant of the *F508del CFTR* (Ile507ATC), that reverts I507 ATT triplet to original ATC found in the wild type sequence, has wild type-like CFTR mRNA structure and enhanced expression levels when compared with native *F508del CFTR* [12]. More importantly, this substitution also affects the function of the protein [13] and sensitivity to drugs [11].

CFTR folding appears to be extremely complex (reviewed in [36]). The slow predicted translation rate for the MSDs makes sense given that homology models predict a complex domain swap structure of two six-spanning helical bundles containing transmembranes 1–2, 9–12 and transmembranes 7–8, 3–6 that are twisted around a central ion-conducting pore [36, 54]. Furthermore, CFTR transmembrane helices contain a number of charged residues which may be important for this complex arrangement of TMDs, and this in combination with the hydrophobic amino acids and the non-optimal codons could slow down translation, and in doing so, provide the necessary time for the proper assembly for this complex, pore-forming structure. Hopefully, this type of *in silico* analysis and discussion provides a framework for where to begin analyzing synonymous polymorphisms and establishes the concept that these types of changes should not be overlooked in future genetic screens.

## Prospects

How codon usage and mRNA structure affect protein translational rates are just beginning to be understood. Algorithms for mRNA structure predictions identify the lowest energy structure among a mixture of structures that certainly exist in equilibrium [45, 55, 56]. Even if the correct structure is identified, supporting biochemical evidence by circular dichroism studies or mRNA folding assays such as the SHAPE assay need to be performed to confirm the predictions [12]. This also means that clonal cell lines have to be established to test for these effects, and the mRNA and protein expression levels and stabilities need to be tested. In the case of I507-ATC- > ATT, we found this synonymous codon change altered the mRNA structure and protein expression levels [12], increased the thermal stability and channel gating properties as monitored whole-cell patch-clamp recordings and single channel recordings, respectively [13], and altered the channel’s sensitivity to drugs [11]. In this particular case, the *I507* sSNP exacerbated the effect of the *F508del* mutation. This suggests the intriguing possibility that other silent polymorphisms have the potential to exacerbate or even mollify disease-causing mutations, or in extreme cases, even be disease-causing themselves. Given the large number of silent polymorphisms found in most genes, and the amount of work required to determine if a polymorphism actually has any effect, bioinformatics approaches such as the ones described here will continue to be an important aspect of future studies that determine which silent polymorphisms alter protein expression and/or function.

## Conclusions

An interesting aspect of these studies is the fact that the individual rates of translation of the different domains of CFTR are predicted to be very different and are consistent with the idea that the domains fold co-translationally. How these sSNPs actually affect the translational kinetics, however, can only be determined experimentally. An intriguing possibility, however, is that sSNPs, especially in combination with known mutations, could either exacerbate or mollify the severity of the mutation through their influence on the translational kinetics of the domain itself or within a domain-domain interface.

## Additional files

Additional file 1: Supplementary methods. (DOCX 36 kb)

Additional file 2: Table S1. SNPs effects on CFTR RSCU The SNPs with significant ∆RSCU values are marked grey. Depending on database coverage the dbSNP database (rs#) and CFTR mutation database identifiers for SNPs were used. (XLSX 30 kb)

Additional file 3: Table S2. SNPs effects on CFTR mRNA secondary structure. The SNPs with significant *p* - values (<0.2) are marked grey. Furthermore, sSNPs with *p* - value *p* < 0.05 in at least one analysis mode and *p* < 0.2 in the second analysis mode were further considered (marked bold) [8, 9]. (XLSX 37 kb)

Additional file 4: Figure S1. Predicted mRNA structures illustrating how the sSNPs affect the local mRNA structure. The wild type sequence is shown in the left panel and sSNP in the right. The structural models were obtained with RNAsnp software, within a 200 nt region centered around the sSNP. (TIF 5393 kb)

Additional file 5: Figure S2. A. Comparison of C-score values distributions calculated for CFTR sSNPs compared to the entire human genome. Median values are marked with solid lines, error bars represent standard deviations. B. Distribution of sSNPs related C-score values within CFTR primary structure, the ones above solid line represent values that were significantly higher than whole genome sSNP mean + SD (6.36866 + 3.701498 = 10.07), whereas the sSNPs with top 25 % C-score values are marked with solid black triangles. The CFTR domain location is marked above the graph. (TIF 1790 kb)

Additional file 6: Table S3. CADD analysis of CFTR SNPs. SNPs that may affect splicing are marked bold. SNPs with C-score significantly higher than whole genome median are marked light grey, while SNPs with highest quartile C-scores are marked dark grey. (XLSX 28 kb)

## References

[1] Kimchi-Sarfaty C, Oh JM, Kim IW, Sauna ZE, Calcagno AM, Ambudkar SV, Gottesman MM (2007). A “silent” polymorphism in the MDR1 gene changes substrate specificity. Science.

[2] Plotkin JB, Kudla G (2011). Synonymous but not the same: the causes and consequences of codon bias. Nat Rev Genet.

[3] Hunt RC, Simhadri VL, Iandoli M, Sauna ZE, Kimchi-Sarfaty C (2014). Exposing synonymous mutations. Trends Genet.

[4] Bali V, Bebok Z. Decoding mechanisms by which silent codon changes influence protein biogenesis and function. Int J Biochem Cell Biol. 2015;64:58–74. 10.1016/j.biocel.2015.03.011 PMC4461553 25817479

[5] Shah K, Cheng Y, Hahn B, Bridges R, Bradbury NA, Mueller DM (2015). Synonymous codon usage affects the expression of wild type and F508del CFTR. J Mol Biol.

[6] Ge WW, Leystra-Lantz C, Sanelli TR, Mclean J, Wen W, Strong W, Strong MJ (2006). Neuronal tissue-specific ribonucleoprotein complex formation on SOD1 mRNA: alterations by ALS SOD1 mutations. Neurobiol Dis.

[7] Nackley AG, Shabalina SA, Tchivileva IE, Satterfield K, Korchynskyi O, Makarov SS, Maixner W, Diatchenko L (2006). Human catechol-O-methyltransferase haplotypes modulate protein expression by altering mRNA secondary structure. Science.

[8] Gartner JJ, Parker SC, Prickett TD, Dutton-Regester K, Stitzel ML, Lin JC, Davis S, Simhadri VL, Jha S, Katagiri N, Gotea V, Teer JK, Wei X, Morken MA, Bhanot UK, Program NCS, Chen G, Elnitski LL, Davies MA, Gershenwald JE, Carter H, Karchin R, Robinson W, Robinson S, Rosenberg SA, Collins FS, Parmigiani G, Komar AA, Kimchi-Sarfaty C, Hayward NK, Margulies EH, Samuels Y (2013). Whole-genome sequencing identifies a recurrent functional synonymous mutation in melanoma. Proc Natl Acad Sci U S A.

[9] Duan J, Wainwright MS, Comeron JM, Saitou N, Sanders AR, Gelernter J, Gejman PV (2003). Synonymous mutations in the human dopamine receptor D2 (DRD2) affect mRNA stability and synthesis of the receptor. Hum Mol Genet.

[10] Reamon-Buettner SM, Sattlegger E, Ciribilli Y, Inga A, Wessel A, Borlak J (2013). Transcriptional defect of an inherited NKX2-5 haplotype comprising a SNP, a nonsynonymous and a synonymous mutation, associated with human congenital heart disease. PLoS One.

[11] Bali V, Lazrak A, Guroji P, Fu L, Matalon S, Bebok Z (2016). A synonymous codon change alters the drug sensitivity of DeltaF508 cystic fibrosis transmembrane conductance regulator. FASEB J.

[12] Bartoszewski RA, Jablonsky M, Bartoszewska S, Stevenson L, Dai Q, Kappes J, Collawn JF, Bebok Z (2010). A synonymous single nucleotide polymorphism in DeltaF508 CFTR alters the secondary structure of the mRNA and the expression of the mutant protein. J Biol Chem.

[13] Lazrak A, Fu L, Bali V, Bartoszewski R, Rab A, Havasi V, Keiles S, Kappes J, Kumar R, Lefkowitz E, Sorscher EJ, Matalon S, Collawn JF, Bebok Z (2013). The silent codon change I507-ATC- > ATT contributes to the severity of the DeltaF508 CFTR channel dysfunction. FASEB J.

[14] Jackson M, Al-Chalabi A, Enayat ZE, Chioza B, Leigh PN, Morrison KE (1997). Copper/zinc superoxide dismutase 1 and sporadic amyotrophic lateral sclerosis: analysis of 155 cases and identification of a novel insertion mutation. Ann Neurol.

[15] Hosler BA, Nicholson GA, Sapp PC, Chin W, Orrell RW, De Belleroche JS, Esteban J, Hayward LJ, McKenna-Yasek D, Yeung L, Cherryson AK, Dench JE, Wilton SD, Laing NG, Horvitz HR, Brown RH (1996). Three novel mutations and two variants in the gene for Cu/Zn superoxide dismutase in familial amyotrophic lateral sclerosis. Neuromuscul Disord.

[16] Calder VL, Domigan NM, George PM, Donaldson IM, Winterbourn CC (1995). Superoxide dismutase (glu100--gly) in a family with inherited motor neuron disease: detection of mutant superoxide dismutase activity and the presence of heterodimers. Neurosci Lett.

[17] Diatchenko L, Slade GD, Nackley AG, Bhalang K, Sigurdsson A, Belfer I, Goldman D, Xu K, Shabalina SA, Shagin D, Max MB, Makarov SS, Maixner W (2005). Genetic basis for individual variations in pain perception and the development of a chronic pain condition. Hum Mol Genet.

[18] Zuker M (2003). Mfold web server for nucleic acid folding and hybridization prediction. Nucleic Acids Res.

[19] Ogurtsov AY, Shabalina SA, Kondrashov AS, Roytberg MA (2006). Analysis of internal loops within the RNA secondary structure in almost quadratic time. Bioinformatics.

[20] Brest P, Lapaquette P, Mograbi B, Darfeuille-Michaud A, Hofman P (2011). Risk predisposition for Crohn disease: a “menage a trois” combining IRGM allele, miRNA and xenophagy. Autophagy.

[21] Kleizen B, Van Vlijmen T, De Jonge HR, Braakman I (2005). Folding of CFTR is predominantly cotranslational. Mol Cell.

[22] Du K, Lukacs GL (2009). Cooperative assembly and misfolding of CFTR domains in vivo. Mol Biol Cell.

[23] Skach WR (2009). Cellular mechanisms of membrane protein folding. Nat Struct Mol Biol.

[24] Riordan JR (2005). Assembly of functional CFTR chloride channels. Annu Rev Physiol.

[25] Collawn JF, Matalon S (2014). CFTR and lung homeostasis. Am J Physiol Lung Cell Mol Physiol.

[26] Collawn JF, Matalon S (2012). The role of CFTR in transepithelial liquid transport in pig alveolar epithelia. Am J Physiol Lung Cell Mol Physiol.

[27] Collawn JF, Lazrak A, Bebok Z, Matalon S (2012). The CFTR and ENaC debate: how important is ENaC in CF lung disease?. Am J Physiol Lung Cell Mol Physiol.

[28] Rowe SM, Miller S, Sorscher EJ (2005). Cystic fibrosis. N Engl J Med.

[29] Thibodeau PH, Richardson JM, Wang W, Millen L, Watson J, Mendoza JL, Du K, Fischman S, Senderowitz H, Lukacs GL, Kirk K, Thomas PJ (2010). The cystic fibrosis-causing mutation deltaF508 affects multiple steps in cystic fibrosis transmembrane conductance regulator biogenesis. J Biol Chem.

[30] Thibodeau PH, Brautigam CA, Machius M, Thomas PJ (2005). Side chain and backbone contributions of Phe508 to CFTR folding. Nat Struct Mol Biol.

[31] Lukacs GL, Verkman AS (2012). CFTR: folding, misfolding and correcting the DeltaF508 conformational defect. Trends Mol Med.

[32] Pranke IM, Sermet-Gaudelus I (2014). Biosynthesis of cystic fibrosis transmembrane conductance regulator. Int J Biochem Cell Biol.

[33] Meacham GC, Lu Z, King S, Sorscher E, Tousson A, Cyr DM (1999). The Hdj-2/Hsc70 chaperone pair facilitates early steps in CFTR biogenesis. EMBO J.

[34] Ward CL, Kopito RR (1994). Intracellular turnover of cystic fibrosis transmembrane conductance regulator. Inefficient processing and rapid degradation of wild-type and mutant proteins. J Biol Chem.

[35] Braakman I, Hoover-Litty H, Wagner KR, Helenius A (1991). Folding of influenza hemagglutinin in the endoplasmic reticulum. J Cell Biol.

[36] Kim SJ, Skach WR (2012). Mechanisms of CFTR Folding at the Endoplasmic Reticulum. Front Pharmacol.

[37] O'Brien EP, Ciryam P, Vendruscolo M, Dobson CM (2014). Understanding the influence of codon translation rates on cotranslational protein folding. Acc Chem Res.

[38] Pechmann S, Frydman J (2013). Evolutionary conservation of codon optimality reveals hidden signatures of cotranslational folding. Nat Struct Mol Biol.

[39] Tsai CJ, Sauna ZE, Kimchi-Sarfaty C, Ambudkar SV, Gottesman MM, Nussinov R (2008). Synonymous mutations and ribosome stalling can lead to altered folding pathways and distinct minima. J Mol Biol.

[40] Sharp PM, Tuohy TM, Mosurski KR (1986). Codon usage in yeast: cluster analysis clearly differentiates highly and lowly expressed genes. Nucleic Acids Res.

[41] Komar AA, Lesnik T, Reiss C (1999). Synonymous codon substitutions affect ribosome traffic and protein folding during in vitro translation. FEBS Lett.

[42] Bonekamp F, Jensen KF (1988). The AGG codon is translated slowly in E. coli even at very low expression levels. Nucleic Acids Res.

[43] Folley LS, Yarus M (1989). Codon contexts from weakly expressed genes reduce expression in vivo. J Mol Biol.

[44] Ren HY, Grove DE, De La Rosa O, Houck SA, Sopha P, Van Goor F, Hoffman BJ, Cyr DM (2013). VX-809 corrects folding defects in cystic fibrosis transmembrane conductance regulator protein through action on membrane-spanning domain 1. Mol Biol Cell.

[45] Sabarinathan R, Tafer H, Seemann SE, Hofacker IL, Stadler PF, Gorodkin J (2013). The RNAsnp web server: predicting SNP effects on local RNA secondary structure. Nucleic Acids Res.

[46] Sabarinathan R, Tafer H, Seemann SE, Hofacker IL, Stadler PF, Gorodkin J (2013). RNAsnp: efficient detection of local RNA secondary structure changes induced by SNPs. Hum Mutat.

[47] Kircher M, Witten DM, Jain P, O'Roak BJ, Cooper GM, Shendure J (2014). A general framework for estimating the relative pathogenicity of human genetic variants. Nat Genet.

[48] Zhang G, Hubalewska M, Ignatova Z (2009). Transient ribosomal attenuation coordinates protein synthesis and co-translational folding. Nat Struct Mol Biol.

[49] Saunders R, Deane CM (2010). Synonymous codon usage influences the local protein structure observed. Nucleic Acids Res.

[50] Ambudkar SV, Kimchi-Sarfaty C, Sauna ZE, Gottesman MM (2003). P-glycoprotein: from genomics to mechanism. Oncogene.

[51] Shen LX, Basilion JP, Stanton VP (1999). Single-nucleotide polymorphisms can cause different structural folds of mRNA. Proc Natl Acad Sci U S A.

[52] Wen JD, Lancaster L, Hodges C, Zeri AC, Yoshimura SH, Noller HF, Bustamante C, Tinoco I (2008). Following translation by single ribosomes one codon at a time. Nature.

[53] Alder NN, Johnson AE (2004). Cotranslational membrane protein biogenesis at the endoplasmic reticulum. J Biol Chem.

[54] Aller SG, Yu J, Ward A, Weng Y, Chittaboina S, Zhuo R, Harrell PM, Trinh YT, Zhang Q, Urbatsch IL, Chang G (2009). Structure of P-glycoprotein reveals a molecular basis for poly-specific drug binding. Science.

[55] Madanecki P, Nozell S, Ochocka R, Collawn JF, Bartoszewski R (2014). RNAdigest: a web-based tool for the analysis and prediction of structure-specific RNAse digestion results. PLoS One.

[56] Wiese KC, Hendriks A (2006). Comparison of P-RnaPredict and mfold--algorithms for RNA secondary structure prediction. Bioinformatics.

[57] Serohijos AW, Hegedus T, Aleksandrov AA, He L, Cui L, Dokholyan NV, Riordan JR (2008). Phenylalanine-508 mediates a cytoplasmic-membrane domain contact in the CFTR 3D structure crucial to assembly and channel function. Proc Natl Acad Sci U S A.

