# Supplementary material for: Codon bias and the folding dynamics of the cystic fibrosis transmembrane conductance regulator
Source: Cell Mol Biol Lett. 2016 Oct 19;21:23. doi: 10.1186/s11658-016-0025-x (PMC5415761; doi:10.1186/s11658-016-0025-x)
Supplement: Supplementary file 1 — Supplementary methods. (DOCX 36 kb) [file 11658_2016_25_MOESM1_ESM.docx]

**Supplemental Materials**

***Codon Bias and the Folding Dynamics of the Cystic Fibrosis Transmembrane Conductance Regulator***

Rafal Bartoszewski (rafalbar@gumed.edu.pl)^1¥*^, Jaroslaw Króliczewski (jarekk@ibmb.uni.wroc.pl)^2¥^, Arkadiusz Piotrowski(arpiotr@gumed.edu.pl)^1^, Anna Janaszak - Jasiecka (ajanaszak@gumed.edu.pl)^1^, Sylwia Bartoszewska (sylwiabart@gumed.edu.pl)^3^; Briana Vecchio-Pagan (briana.vecchio@gmail.com)^4^ , Lianwu Fu (lianwufu@uab.edu)^6,8^, Aleksandra Sobolewska (aleksandrasobolewska@gumed.edu.pl)^1^, Sadis Matalon (sadis@uab.edu)^5,6,8^, Garry R. Cutting (gcutting@jhmi.edu)^4^, Steven M. Rowe (SRowe@peds.uab.edu)^6,7,8^, and James F. Collawn (jcollawn@uab.edu) ^6,8¥^

**Methodology**

***Codon usage***. The relative synonymous codon usage method (RSCU) was used in order to calculate potential codon impact on the translational rate of CFTR [[1-4](#_ENREF_1)]. Briefly, we calculated RSCU values for each CFTR codon as RSCU = SN_C_/N_A_, where N_C_ is the frequency of particular codon "C" within the human genome, while N_A_ depicts frequency of amino acid ("A") encoded by the codon "C". Since all amino acids except methionine and tryptophan can be also encoded by more than one codon, S refers to the number of synonymous codons for each amino acid. The frequencies were obtained from the Codon Usage Database [[5](#_ENREF_5), [6](#_ENREF_6)]. In order to adapt the RSCU method for analysis of CFTR domains and structural motifs, and to determine the most significant changes in RSCU introduced by the sSNPs, each RSCU value was expressed as a logarithm base 10 for each codon and the two neighboring codons (RSCU_logM_). These “transformed-moving-median-logarithm values” reduce the statistical impact of single codons, and allow for selecting the primary structural motifs that may have an optimal (fast) or suboptimal (slow) translation rate. Taking the log provides a result centered around zero, where a slower than average codon is negative, while a faster is positive [[7](#_ENREF_7)].

***CFTR sSNPs selection.*** The analyzed CFTR sSNPs were obtained by combining data from Cystic Fibrosis Mutation Database (CFTR1; <http://www.genet.sickkids.on.ca/app>) and The Single Nucleotide Polymorphism Database (dbSNP) of Nucleotide Sequence Variation (NCBI) (<http://www.ncbi.nlm.nih.gov/SNP>). Only sSNPs within CFTR mRNA sequence that did not result in substitution, deletion, or frameshift were considered in these analyses. NCBI nomenclature was used for the sSNP position mapping.

***RNA structural analysis.*** To determine if the sSNPs induced predicted changes in local CFTR secondary mRNA structures, we applied RNAsnp software [[8](#_ENREF_8), [9](#_ENREF_9)]. The SNPs that were predicted to affect the CFTR mRNA secondary structures were selected in two analysis modes (using 200 nt and 800 nt SNP neighboring sequences) based on their p - value (p<0.05 in at least one analysis mode and p < 0.2 in the second analysis mode) [[8](#_ENREF_8), [9](#_ENREF_9)]. Only sSNPs that were reported as being significant by both sequence lengths were considered further.

***CADD analysis***. To assess the relative pathogenicity of the CFTR's sSNPs, we applied an integrated method for estimating the relative pathogenicity of genetic variants (combined annotation–dependent depletion (CADD)) based on annotations from a variety of sources and combining them into a single measure that is expressed as C-score (as described in [[10](#_ENREF_10)]) Here, the C-score ranks based on CADD annotations were used to aid in estimating the significance of the sSNP mutations against a backdrop of the gene polymorphism spectrum in the general population. The detected mutations were placed in the context of known variants in the affected genes from 1KG and ESP6500SI-V2 by assigning the (CADD) C-score to each variant, and presenting the C-score rank of mutations in scatter plots.

***Statistics***

The Shapiro-Wilk [[11](#_ENREF_11)] or D'Agostino-Pearson [[12](#_ENREF_12)] normality tests were used to test for a normally distributed population. The Shapiro-Wilk test works very well if every value is unique, whereas it is not as effective when several values are identical. In those instances, the D'Agostino-Pearson test was used instead [[12](#_ENREF_12)]. Statistical analyses of variance were made using ANOVA followed by a Post Hoc Test [[13](#_ENREF_13), [14](#_ENREF_14)]. Multiple comparisons were performed only when ANOVA p-values were significant. The p-value was calculated under the null hypothesis that the samples were drawn from the same distribution. Statistical significance for ANOVA was accepted at a p-value less than 0.05.

The analyses were performed with Statistica version 10.0 (StatSoft Inc., Tulsa, OK, USA) and R software (R Foundation Statistical Computing, AT). R is an open source project that is distributed under the GNU General Public License (Copyright 2007 Free Software Foundation, Inc.) [[15](#_ENREF_15)]. The t-test was used to verify if the sSNPs altered the predicted translational rate of CFTR.

**Figure legends**

**Supplemental Figure 1.** Predicted mRNA structures illustrating how the sSNPs affect the local mRNA structure. The wild type sequence is shown in the left panel and sSNP in the right. The structural models were obtained with RNAsnp software, within a 200 nt region centered around the sSNP.

**Supplemental Figure 2. A** Comparison of C-score values distributions calculated for CFTR sSNPs compared to the entire human genome. Median values are marked with solid lines, error bars represent standard deviations. **B.** Distribution of sSNPs related C-score values within CFTR primary structure, the ones above solid line represent values that were significantly higher than whole genome sSNP mean + SD (6.36866 + 3.701498 = 10.07), whereas the sSNPs with top 25% C-score values are marked with solid black triangles. The CFTR domain location is marked above the graph.

**Supplemental Table 1.** SNPs effects on CFTR RSCU The SNPs with significant ∆RSCU values are marked grey. Depending on database coverage the dbSNP database (rs#) and CFTR mutation database identifiers for SNPs were used.

**Supplemental Table 2.** SNPs effects on CFTR mRNA secondary structure. The SNPs with significant p - values (<0.2) are marked grey. Furthermore, sSNPs with p - value p<0.05 in at least one analysis mode and p < 0.2 in the second analysis mode were further considered (marked bold). [[8](#_ENREF_8), [9](#_ENREF_9)]

**Supplemental Table 3.** CADD analysis of CFTR SNPs. SNPs that may affect splicing are marked bold. SNPs with C-score significantly higher than whole genome median are marked light grey, while SNPs with highest quartile C-scores are marked dark grey.

**References**

1 Sharp, P. M., Tuohy, T. M., Mosurski, K. R. 1986 Codon usage in yeast: cluster analysis clearly differentiates highly and lowly expressed genes. *Nucleic Acids Res*. **14**, 5125-5143.

2 Komar, A. A., Lesnik, T., Reiss, C. 1999 Synonymous codon substitutions affect ribosome traffic and protein folding during in vitro translation. *FEBS Lett*. **462**, 387-391.

3 Bonekamp, F., Jensen, K. F. 1988 The AGG codon is translated slowly in E. coli even at very low expression levels. *Nucleic Acids Res*. **16**, 3013-3024.

4 Folley, L. S., Yarus, M. 1989 Codon contexts from weakly expressed genes reduce expression in vivo. *J Mol Biol*. **209**, 359-378.

5 Nagase, T., Kikuno, R., Ohara, O. 2003 The Kazusa cDNA project for identification of unknown human transcripts. *C R Biol*. **326**, 959-966.

6 Sauna, Z. E., Kimchi-Sarfaty, C. 2011 Understanding the contribution of synonymous mutations to human disease. *Nat Rev Genet*. **12**, 683-691. (10.1038/nrg3051)

7 Saunders, R., Deane, C. M. 2010 Synonymous codon usage influences the local protein structure observed. *Nucleic Acids Res*. **38**, 6719-6728. (10.1093/nar/gkq495)

8 Sabarinathan, R., Tafer, H., Seemann, S. E., Hofacker, I. L., Stadler, P. F., Gorodkin, J. 2013 The RNAsnp web server: predicting SNP effects on local RNA secondary structure. *Nucleic Acids Res*. **41**, W475-479. (10.1093/nar/gkt291)

9 Sabarinathan, R., Tafer, H., Seemann, S. E., Hofacker, I. L., Stadler, P. F., Gorodkin, J. 2013 RNAsnp: efficient detection of local RNA secondary structure changes induced by SNPs. *Hum Mutat*. **34**, 546-556. (10.1002/humu.22273)

10 Kircher, M., Witten, D. M., Jain, P., O'Roak, B. J., Cooper, G. M., Shendure, J. 2014 A general framework for estimating the relative pathogenicity of human genetic variants. *Nat Genet*. **46**, 310-315. (10.1038/ng.2892)

11 Shapiro, S. S. W., M. B. 1965 An analysis of variance test for normality (complete samples). *Biometrika*. **52**, 591-611.

12 D'Agostino, R. B., Belanger, A., D'Agostino, R. B. 1990 A Suggestion for Using Powerful and Informative Tests of Normality. *The American Statistician*. **44**, 316-321. (10.1080/00031305.1990.10475751)

13 Hommel, G. 1988 A stagewise rejective multiple test procedure based on a modified Bonferroni test. . *Biometrika*. **75**, 383-386.

14 Holm, S. 1979 A simple sequentially rejective multiple test procedure. *Scandinavian Journal of Statistics*. **6**, 65-70.

15 R Core Team. R: A Language and Environment for Statistical Computing.: R Foundation for Statistical Computing, Vienna, Austria (2013) 2013.
