# Supplementary figures and images for: Codon bias and the folding dynamics of the cystic fibrosis transmembrane conductance regulator
Source: Cell Mol Biol Lett. 2016 Oct 19;21:23. doi: 10.1186/s11658-016-0025-x (PMC5415761; doi:10.1186/s11658-016-0025-x)

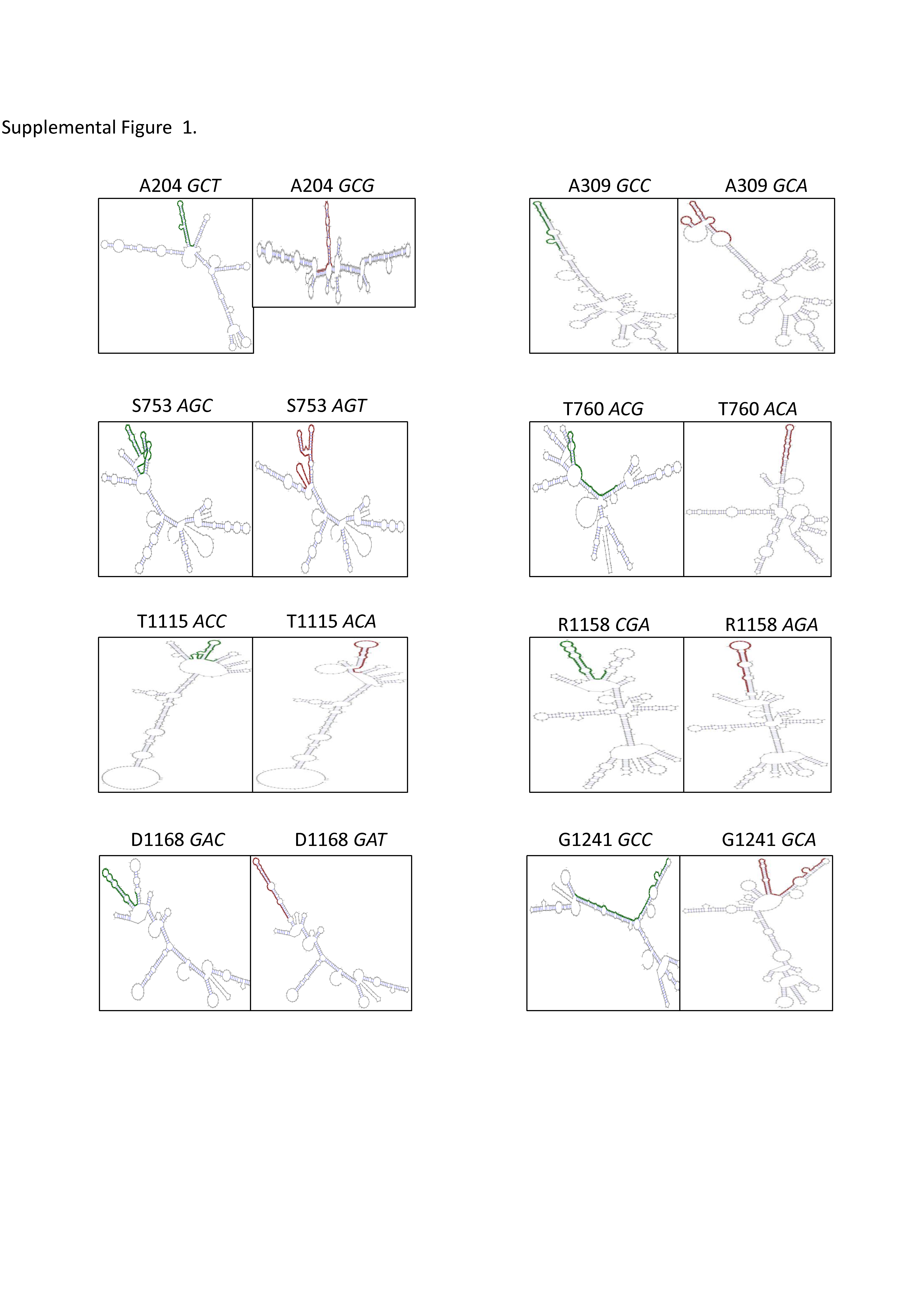

Supplement: Supplementary file 4 — Predicted mRNA structures illustrating how the sSNPs affect the local mRNA structure. The wild type sequence is shown in the left panel and sSNP in the right. The structural models were obtained with RNAsnp software, within a 200 nt region centered around the sSNP. (TIF 5393 kb) [file 11658_2016_25_MOESM4_ESM.tif]

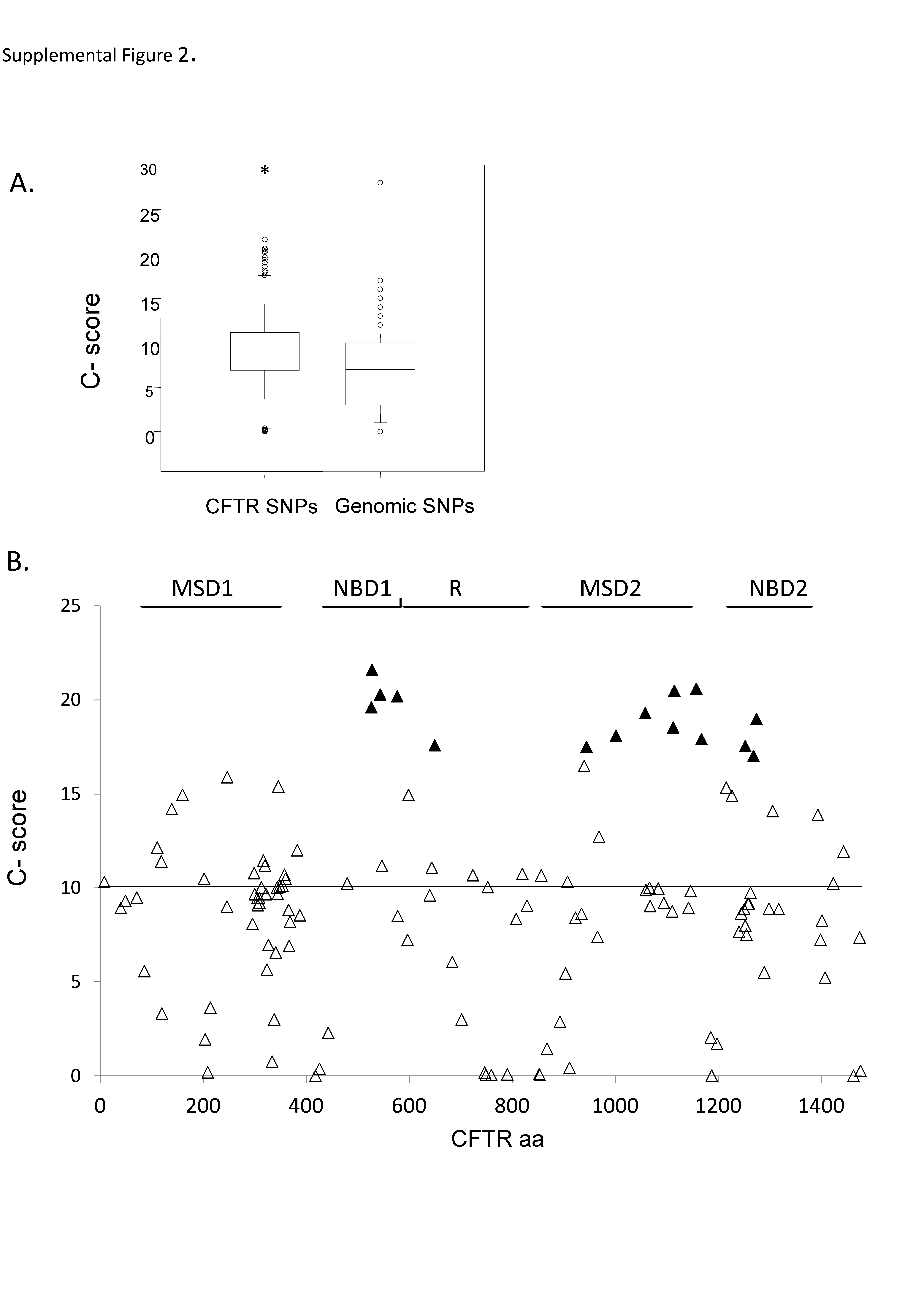

Supplement: Supplementary file 5 — A. Comparison of C-score values distributions calculated for CFTR sSNPs compared to the entire human genome. Median values are marked with solid lines, error bars represent standard deviations. B. Distribution of sSNPs related C-score values within CFTR primary structure, the ones above solid line represent values that were significantly higher than whole genome sSNP mean + SD (6.36866 + 3.701498 = 10.07), whereas the sSNPs with top 25 % C-score values are marked with solid black triangles. The CFTR domain location is marked above the graph. (TIF 1790 kb) [file 11658_2016_25_MOESM5_ESM.tif]
